# Site-fidelity and spatial movements of western North Pacific gray whales on their summer range off Sakhalin, Russia

**DOI:** 10.1371/journal.pone.0236649

**Published:** 2020-08-14

**Authors:** Koen C. A. Bröker, Glenn Gailey, Olga Yu. Tyurneva, Yuri M. Yakovlev, Olga Sychenko, Jennifer M. Dupont, Vladimir V. Vertyankin, Evgeny Shevtsov, Konstantin A. Drozdov

**Affiliations:** 1 Marine Evolution and Conservation, Groningen Institute for Evolutionary Life Sciences, University of Groningen, Groningen, the Netherlands; 2 Shell Global Solutions International B.V., the Hague, The Netherlands; 3 Cetacean EcoSystem Research, Washington, Olympia, United States of America; 4 A.V. Zhirmunsky National Scientific Center of Marine Biology of the Far Eastern Branch of the Russian Academy of Sciences (NSCMB FEB RAS), Vladivostok, Russian Federation; 5 ExxonMobil Upstream Research Company, Houston, Texas, United States of America; 6 Kronotsky State Biosphere Reserve, Elizovo, Russian Federation; 7 G.B. Elyakov Pacific Institute of Bioorganic Chemistry of the Far Eastern Branch of the Russian Academy of Sciences (PIBOC FEB RAS), Vladivostok, Russian Federation; Brigham Young University, UNITED STATES

## Abstract

The Western North-Pacific (WNP) gray whale feeding grounds are off the northeastern coast of Sakhalin Island, Russia and is comprised of a nearshore and offshore component that can be distinguished by both depth and location. Spatial movements of gray whales within their foraging grounds were examined based on 13 years of opportunistic vessel and shore-based photo-identification surveys. Site fidelity was assessed by examining annual return and resighting rates. Lagged Identification Rates (LIR) analyses were conducted to estimate the residency and transitional movement patterns within the two components of their feeding grounds. In total 243 individuals were identified from 2002–2014, among these were 94 calves. The annual return rate over the period 2002–2014 was 72%, excluding 35 calves only seen one year. Approximately 20% of the individuals identified from 2002–2010 were seen every year after their initial sighting (including eight individuals that returned for 13 consecutive years). The majority (239) of the WNP whales were observed in the nearshore area while only half (122) were found in the deeper offshore area. Within a foraging season, there was a significantly higher probability of gray whales moving from the nearshore to the offshore area. No mother-calf pairs, calves or yearlings were observed in the offshore area, which was increasingly used by mature animals. The annual return rates, and population growth rates that are primarily a result of calf production with little evidence of immigration, suggest that this population is demographically self-contained and that both the nearshore and offshore Sakhalin feeding grounds are critically important areas for their summer annual foraging activities. The nearshore habitat is also important for mother-calf pairs, younger individuals, and recently weaned calves. Nearshore feeding could also be energetically less costly compared to foraging in the deeper offshore habitat and provide more protection from predators, such as killer whales.

## Introduction

The Eastern (ENP) and Western North Pacific (WNP) gray whales (*Eschrichtius robustus*) are the only known extant populations of this species. The North Pacific populations were significantly depleted during early- to mid-20th century commercial whaling, while the Atlantic gray whales were hunted to extinction a few hundred years earlier [1–3]. The WNP whale population was presumed extinct by 1966 [4], but small numbers of gray whales were sighted in the late 1960s and 1970s within the Sea of Okhotsk, South China Sea and the Sea of Japan [5–7]. Meanwhile, the ENP gray whale population recovered to close to 21,000 individuals from 1985 onwards [8–10], with indications that this population may have reached carrying capacity [11]. The WNP population is recovering at a much slower pace. Pre-exploitation abundance of this population was estimated to be between 1,500–10,000, with 1,000–1,500 individuals remaining in the population in 1910 after commercial exploitation had started [5, 12, 13]. In 2015, the non-calf WNP population numbered ca. 174–186 individuals [14]. Due to the low population size, the WNP population is listed as endangered in the Red Data Book of the Russian Federation [15]. The IUCN red list status of the WNP population is currently endangered and was updated from critically endangered in 2018 [16–18].

The main known WNP summer feeding areas are situated in the Sea of Okhotsk [19]. WNP gray whales have been sighted during the summer off northeast Sakhalin in two main feeding areas: (1) the nearshore waters where whales predominantly feed in shallow waters (<20 m depth) [20] and (2) the offshore waters where whales feed in deeper water depths (35–60 m) [21–27]. Based on whaling catch data, it was assumed that WNP whales winter at the southern end of the Korean peninsula [28]. Other historic records suggests these wintering grounds may be as far south as the Yellow Sea, East China Sea and South China Sea [2]. Satellite-tags deployed on WNP individuals [29, 30] and genetic and photo-ID comparisons between ENP and WNP individuals [31–35] demonstrated that at least a portion of the WNP whales migrated to Baja California. This indicated some degree of spatial overlap between the two populations, as ENP whales typically migrate from breeding grounds near Baja California to summer foraging areas in the Bering and Chukchi Seas [36–39]. However, genetic studies have consistently found significant differentiation, with some limited genetic exchange occurring [31, 32, 38, 40–44]. One key difference between the WNP feeding grounds off Sakhalin versus those used by ENP gray whales, is the spatial area of these feeding grounds. With approximately 600 and 700 km^2^ for the nearshore and offshore feeding areas, respectively, the feeding grounds used by WNP whales [45] are drastically smaller in comparison to those used by ENP whales. A tagging study on 23 individuals in the Pacific Coast Feeding Group (PCFG), a subset of ENP whales who do not migrate to the Bering, Chukchi or Beaufort seas to feed, showed individual feeding-area home ranges (90% isopleth) and core areas (50% isopleth) of 3,107 km^2^ (±4,140) and 840 km^2^ (±1,159), respectively [46]. Home ranges covered most of the near-shore waters from Northern California to Icy Bay, Alaska. Core areas showed a similar range and overlapped for multiple whales in some areas so the total area available for foraging by PCFG whales was significantly larger than the mean individual core area. Similar results were also found for tagged ENP gray whales off Chukotka, Russia, that had a mean core area of 2,087 km^2^ [36]. The relatively small spatial scale of the WNP whale feeding grounds can makes these whales more susceptible to environmental perturbations, such as seasonal ice cover duration [45], or anthropogenic disturbance [47, 48], as there are no other known feeding areas in the area. As capital breeders, gray whales primarily rely on the acquisition of food resources during their summer feeding season to sustain them through their migration and breeding/calving season [49–51], with estimated individual gray whale consumption of about 409 kg of benthic prey per day, or ~61 tons during the 5-month feeding period along Chukotka, Russia [52]. As bottom-feeding specialists, gray whales feed on benthic and epi-benthic organisms and ingest their food by suction [2, 52, 53]. In the nearshore feeding area off Sakhalin, the benthic community is predominately comprised of amphipods (primarily *Monoporeia affinis*), isopods, bivalves, cumaceans, epibenthic crustaceans and sand lance (*Ammodytes hexapterus*) with amphipods presumably being preferential prey for gray whales due to their high caloric content, dense populations and high biomass [52, 54, 55]. While the nearshore area functions as habitat for smaller/shorter lived amphipods, the offshore feeding area contains dense aggregations of Amplelisca amphipods *Ampelisca eschrichtii* which are much larger and longer lived. Based on stomach content analyses in most other parts of the world these have been identified to be the primary prey for gray whales [11, 13, 21, 22, 52, 56, 57]. Annual prey resource studies off Sakhalin found that biomass of amphipods in the nearshore feeding area ranged from higher biomass at shallower depths (93.0 g/m^2^ at 10-15m depths in 2012) to lower biomass in deeper waters (19.9 g/m^2^ at 21-25m in 2012) [57]. In comparison, the mean amphipod biomass in the deeper offshore feeding area (338.2 g/m^2^) was consistently higher [21, 57]. These differences in biomass between the nearshore and offshore feeding areas could be one explanatory factor for some of the observed spatial movement patterns of the WNP population within their foraging grounds [21, 24, 57].

Gray whales are known to display maternally directed site fidelity, which is also observed in other baleen whales like Humpback whales (*Megaptera novaeangliae*) [58]. For example, humpback whales follow seasonal migration cycles between high-latitude feeding grounds and low-latitude breeding regions as well [59]. Photo-identification studies of humpback whales conducted in the North Pacific and North Atlantic indicated strong site fidelity to feeding areas with high rates of resightings in the same feeding area, and very limited interchange between different feeding areas [59–61]. In the North Pacific this was even the case when feeding areas were in relatively close proximity (<100km) [59], which appeared different from animals in the North Atlantic that make longer daily feeding trips (>100km) [62]. Gray whales, however, tend to display more focused foraging efforts on a relatively smaller spatial scale with higher coastal affinity [36, 62–67], compared to some other species of baleen whales. This may be explained by presence of sufficient prey availability without the need to make long or rapid movement to other areas. (Epi)benthic feeding baleen whales, such as gray whales, but also bowheads whales, are thought to encounter more predictable, but spatially restricted prey concentrations [68], than other pelagic fish or krill feeding baleen whales [36, 69]. Spatial movement patterns between the two feeding areas off Sakhalin, as well as site fidelity to these areas, are currently not well defined.

In this study, we quantified the WNP gray whale annual site fidelity and residence and examined differences in their utilization of the two components of their feeding grounds off Sakhalin based on a long-term (13-year) photo-identification dataset. Annual return and resighting rates were used as metrics of site fidelity. Residency, defined as the time spent by an animal in a specific geographical area [70, 71], has not been previously determined for WNP gray whales on their feeding grounds. Lagged Identification Rates were used to assess mean residence time on their foraging grounds off Sakhalin as well as within and between the nearshore and offshore feeding areas of their feeding habitat.

## Materials and methods

### Study area

**Fig 1:**
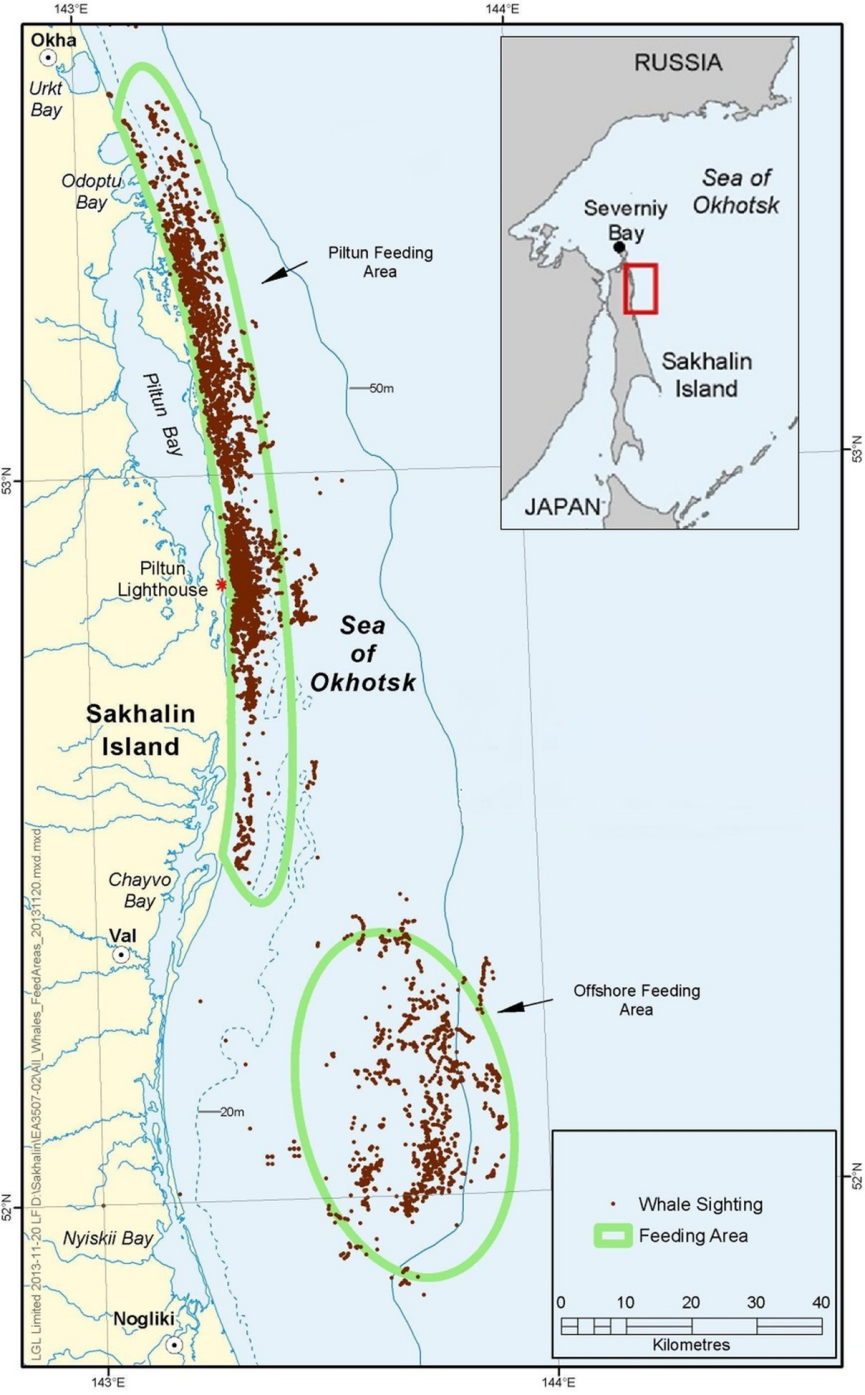
Photo-identification study areas off Sakhalin Island, Russia. Locations of gray whale sightings in the study area are designated as brown dots. Top right inset shows a regional view of the northeast Sakhalin Island.

Annual opportunistic photo-identification surveys of gray whales were conducted in the waters off northeast Sakhalin Island from 2002–2014. The survey areas covered the known nearshore and offshore feeding areas. The nearshore area was approximately 120 km along the shore adjacent to the Piltun and Chayvo lagoons (52.3° - 53.3°N), approximately up to the 20 m contour (~5–10 km from shore, with peak densities between 500 and 2000 m [72]), with the mouth of the Piltun lagoon located towards the middle. Sightings made within this area were considered nearshore, but also included a limited number of sightings beyond 10 km from shore. The offshore feeding area was mostly south of Chayvo lagoon and approximately 30–40 km from the coast, with depths of 40–50 m (Fig 1). Sightings between 51.8° and 52.4° N and 143.4° and 144.0° E were classified as offshore. Substrates in the nearshore areas include silty sands with more sandy muds in the offshore area [73]. Both feeding areas have amongst the highest primary and secondary productivity in the Sea of Okhotsk [21].

### Photo-identification surveys

During the period 2002–2014, opportunistic photo-identification effort in the nearshore and offshore areas off NE Sakhalin was conducted from either an inflatable boat (4.8 m) or a larger research vessel. No predetermined systematic survey tracks were followed. Surveys were executed to maximize both spatial coverage as well as encountering and photographically identifying as many gray whales as possible. Marine mammal observers on the research vessel scanned the feeding areas for the presence of gray whales. Upon sighting gray whales an inflatable boat was deployed from the larger research vessel to photographically capture the sighted whales. Animals sighted near the larger research vessel were also photographically captured if conditions allowed. However, this platform was not used to actively approach individuals or groups of gray whales. Additionally, opportunistic, shore-based, photo-identification efforts were implemented from 2004 to 2010 from various observation stations with height ranging from 5 to 30 m. Although maximum observation distance was 10–40 km from shore, the maximum distance for identifying individuals was limited to 1–3 km, depending on atmospheric conditions, due to limitations of capturing animals with photographic equipment. In 2014 vehicle-based surveys were conducted from the beach during which animals were observed close to shore. In 2014, photo-ID research effort in the nearshore area was expanded to include an additional inflatable boat that was launched from within the Piltun lagoon.

Every observation of a gray whale group was recorded if one or more individuals from that group were photographed, with a sighting being defined as the capture of a single individual. There could be several sightings per survey. A group was defined as either a single animal, or multiple gray whales swimming in proximity to each other (within 10 body lengths) with coordinated behavior relative to other individuals. The inflatable boat team consisted of a boat driver, data recorder, video camera operator, and camera photographer. Whales were slowly approached from the side, with a no-approach zone of 100 m. Individuals were then photographed to capture identifiable features that included the head, fluke, and sides of their body. Priority was given to the right dorsal flank, followed by the left dorsal flank, ventral and dorsal aspects of the fluke. The date, time, camera frame numbers for each individual whale, group size, geographic information (e.g. distance to the whale(s), observer location (via GPS), and environmental parameters such as Beaufort sea state and air temperature were recorded by both vessel- and shore-based photo-identification teams. Vessel-based teams recorded water depth and water temperature as well. A range of cameras were used (Canon (7D), Nikon (D1X, D2X, DF100, DF700). Vessel-based teams used 70–400 and fixed 300 mm lenses, while the shore-based teams used lenses with extended range from 70–800 mm with up to 2x extenders.

### Individual-matching methodology

The field methodology for the photo-identification surveys and matching procedures followed Hammond *et al*. [74] with modifications specially aimed at gray whales [20, 75, 76]. Distinctive marks on sides and flukes of gray whales were used to identify individual animals [74]. The primary feature for identifying individuals was body pigmentation, whereby scars and barnacle patches could provide additional matching confirmation [76]. The left, right and fluke identifiable aspects of the individual were recognized based on following criteria: (1) the whale was photographed as a solitary individual; (2) photographic series of both sides of the whale with the same fluke were obtained during one sighting; (3) the height, spacing, and ratio of the distinctive knuckles in the ridge on the caudal peduncle were considered as a final check. Identifications and right to left matches were verified by at least two independent matching specialists. Only excellent and good quality photographs were used for matching individuals and right to left matching to avoid false negatives, unless individuals were highly distinctive on images of poorer quality [77].

Calves usually arrive on the foraging grounds accompanied by their mothers. During the course of their feeding season calves are weaned from their mothers. The weaning commonly occurs from August to mid-September. Criteria for identification of calves and presumed mothers were used that are described in Yakovlev and Tyuerneva [78] and included morphological and behavioural features distinctive to calves such as presence of vibrissae, separation distance, as well as the number of sightings with a candidate mother. New individuals were categorized as either calves, i.e. animals meeting the criteria for recently born individuals, or non-calves, i.e. individuals entering the Sakhalin population from elsewhere or individuals that were missed as a calf.

After finalization of the matching process, the highest quality sighting images in terms of clarity, contrast, content and angle [77], were stored in the photo-identification database software ‘Discovery’ [79], together with environmental conditions, sighting location, and other sighting information.

### Data analyses

Because animals were sighted from multiple observation platforms (i.e. inflatable boat, research vessel, shore-based platforms), effort was determined by using the cumulative number of all sightings made over the season. Differences in nearshore and offshore area usage was assessed by comparing the number of individuals observed in these areas each year, after standardizing the number of seasonal sightings in both areas, using a Chi-squared goodness-of-fit test. For the period 2003–2014 more sightings were made in the nearshore area. Therefore, the number of sightings made in the offshore area was assessed for each year, and consequently the same number of sightings was randomly sampled with replacement from the total pool of nearshore sightings for that same year. The number of unique individuals in this random sample of sightings was then determined. In 2002, more annual sightings were made in the offshore area. The same number of sightings made in the nearshore area in 2002 was therefore randomly sampled with replacement from the total pool of offshore area sightings made in that year. The total number of unique individuals was consequently assessed from that sample of sightings. Discovery curves, i.e. the cumulative number of unique individuals as a function of survey effort, expressed by the cumulative number of sightings, were generated for new individuals, new individuals that were not calves, and new individuals that were calves.

Site fidelity can be defined as the tendency of an animal to remain in an area over an extended period or to return to an area previously occupied [80]. This area-restricted space use behavior has important consequences for many ecological processes [81] and can be estimated by using the repeated presence of individual whales in a feeding areas over time [59]. On a population level, site fidelity can also reflect the annual return rate [59]. Site fidelity to the Sakhalin feeding grounds was inferred from two different statistics. The first used metric was the annual return rate, which is was defined as the number of individuals re-sighted each year after the observation of the total number of individuals for the first time in a given year. The second metric was the annual re-sighting rate, which is defined as the number of individuals sighted in the current year that were identified in previous years, divided by the total number of animals sighted that year [59].

Mean residency times were defined as the time spent by an animal in a specific geographical area [70, 71]. Residency times in the study area were estimated by using the Lagged Identification Rate (LIR) [82, 83]. LIR (*R*(*τ*)) is defined as the probability of identifying a random individual in an area at time (t) = 0, and re-sighting that same individual again after a variable lag time (*τ*) (t = 0 + *τ*) [82]. *R*(*τ*) is the probability that an individual in a study area at time 0 is also in at after a lag time of *τ* (*P*(*τ*)), divided by the number of individuals (N) in the study area:

R(τ)=P(τ)/N

For any lag time (*τ*), *R*(*τ*) can be estimated from the proportions of the total pairs of identification *τ* time units apart (*g*(*τ*)), which are of the same individual (*m*(*τ*)):

$$\hat{R}(\tau )=m(\tau )/g(\tau )$$

where

$$m(\tau )=\sum_{i,j}\left\{m_{ij}|\tau_{ij}=\tau \right\}$$

and

$$g(\tau )=\sum_{i,j}\left\{n_{i}\times n_{j}|\tau_{ij}=\tau \right\}$$

In these formulas, _i, j_ are used to denote a set of individual identifications collected at a particular time and location, m_ij_ are the number of individuals identified in both set _i_ and set _j_, *τ*_ij_ is the time lag between identification sets _i_ and _j_, and n_i_ and n_j_ are the number of individuals identified in _i_th and _j_th set, respectively.

LIR plotted over time provides insight in the use of a study area by individuals as the LIR remains constant if a population is closed and identifications are independent. In this case, the LIR is the inverse of the population size. The LIR can decrease with increasing time lag due to emigration and mortality. Non-zero LIR levels indicates that some individuals remain resident or that emigrated individuals re-immigrate into the study area [82]. Changes in the LIR over the study period were modeled for both all individuals, as well as for animals first seen as a calf, using maximum likelihood methods in the program SOCPROG 2.8 for the period 2002–2014 [83]. Lagged identification times were binned in time lags of increasing duration, and the LIR was calculated for each bin. Consequently, SOCPROG fits models of lagged identification rates using maximum likelihood and binomial loss by applying the full data set, not simply the estimated lagged identification rates [83]. A set of 8 models populated with preset parameters (S1 Table) was applied to test for closed and open population models, including various combinations of emigration, reimmigration and mortality, and was used to test the empirical dataset [83]. These models were of the exponential form with up to three parameters for processes such as emigration/mortality, mean residency times, mean time out of study area etc. (S1 Table). The quasi-Akaike information criterion (QAIC) was used to evaluate each model’s goodness of fit and account for over-dispersion of data [84].

LIR were also used to examine the transitional probabilities of movement between the offshore and nearshore area, and to estimate the distribution ratio of the Sakhalin population over these two feeding grounds. Four models, also populated with preset parameters, were used to test the migration rates and mean residence periods between the nearshore and offshore feeding areas (S1 Table). Both the entire study area and between feeding areas were assessed for the duration of one feeding season as well as for the overall observation period (2002–2014). Transitional probabilities of movement between the offshore and nearshore area using LIR were also established for animals that were first seen as a calf only. The quasi-Akaike information criterion (QAIC) was used to evaluate each model fit and account for over-dispersion of data [84]. In all the selected models, the 95% confidence interval was estimated by bootstrapping from 100 replicates [85].

### Ethics statement

Due to the non-invasive techniques employed in this study, no permits were required for the field observations of gray whales of Sakhalin. Animals were approached in line with the IWC general principles for whale watching. Protocols and methodology for field observations were reviewed by the Western Gray Whale Advisory Panel (WGWAP), established by the International Union for the Conservation of Nature (IUCN), on an annual basis.

## Results

Photo-identification efforts were concentrated in the nearshore area, including the nearshore waters off Chayvo lagoon in the south, with additional dedicated survey effort in the offshore area (Table 1). Total survey effort in both feeding areas was not equal with more survey effort in the nearshore area since more animals were initially observed there. The nearshore survey effort was mostly focused around the lagoon mouth due to the consistent presence of large number of individuals, but surveys were also conducted in the northern and southern parts of the nearshore feeding area (Fig 1) as substantial inter-annual differences in distribution were observed.

**Table 1:** Summary of photo-identification survey periods, effort, and number of KOGW whales identified for each year off Sakhalin.

Year	Survey Dates	No. of teams	No. of obs. days	Total no. of sightings	No. of Ind.	Resighted ind.	New Ind.	Calves	New non-calf	Total in Catalog
2002	14Sep-15Oct	1/0/0	13	77	49	0	49	0*	49*	49
2003	07Aug-19Sep	1/0/0	26	177	86	38	48	10	38	97
2004	31Jul-01Oct	1/1/0	35	265	101	79	22	3	19	119
2005	13Jul-01Oct	1/1/0	48	502	117	100	17	4	13	136
2006	01Jul-09Oct	1/1/0	51	518	122	110	12	5	7	148
2007	23Jun-05Oct	1/1/0	74	851	127	113	14	10	4	162
2008	01Jul-03Oct	1/1/0	44	317	102	97	5	5	0	167
2009	05Jul-23Sep	1/1/0	42	429	122	111	11	8	3	178
2010	06Jun-27Sep	1/1/0	55	482	121	112	9	7	2	187
2011	19Aug-05Oct	1/0/0	27	351	123	105	18	15	3	205
2012	13Aug-5Oct	1/0/0	33	371	144	130	14	9	5	219
2013	10Jul-11Oct	1/0/0	12	204	121	112	9	6	3	228
2014	26Jul-03Oct	1/2/1	49	696	138	123	15	12	3	243
Total			509	5240	1473		243	94	149	

No. of teams = number of teams per platform (no. of vessel-inflatable boat-based teams / no. of shore-based teams / no. of shore-based inflatable boat teams); No. of obs. days = days with survey effort; ‘Total no. of sightings’ = number of individuals observed, includes multiple sightings per individual; ‘No. of Ind.’ = number of unique individuals; ‘Resighted Ind.’ = individuals that were observed in previous years; ‘New. Ind.’ = number of individuals observed off Sakhalin for the first time;

* = no calves were identified in 2002.

The number of individual whales (and calves) identified each year depended on the number of whales in the area, location, and timing and duration of photo ID effort. There was variation in the annual survey dates and duration (Table 1).

**Fig 2:**
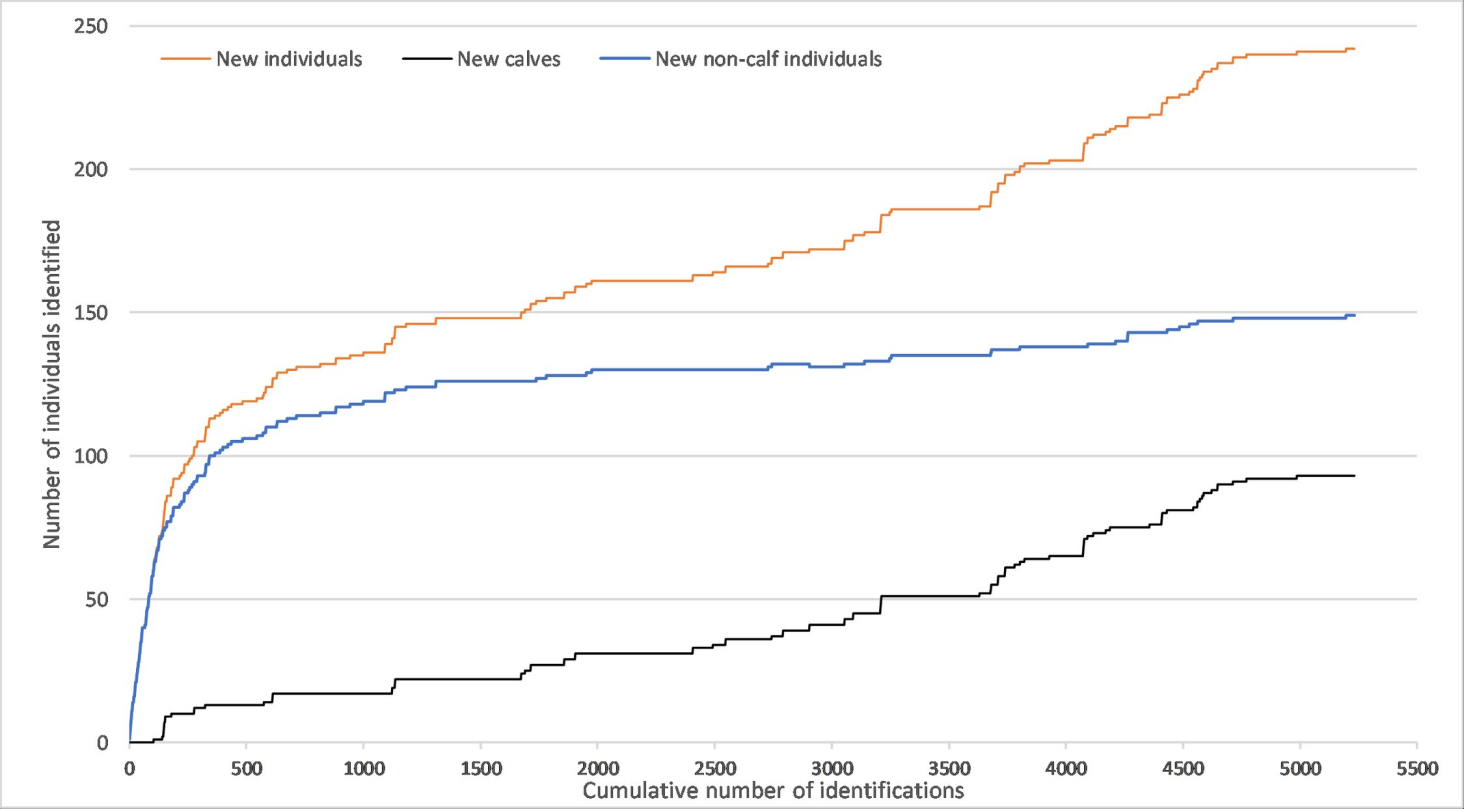
A discovery curve representing the total number of new individuals, number of new non-calves and new calves identified as function of the total cumulative number of identifications made from 2002–2014 in the Sakhalin feeding grounds. No distinction was made between calves and non-calves during the first year of the study (2002).

**Fig 3:**
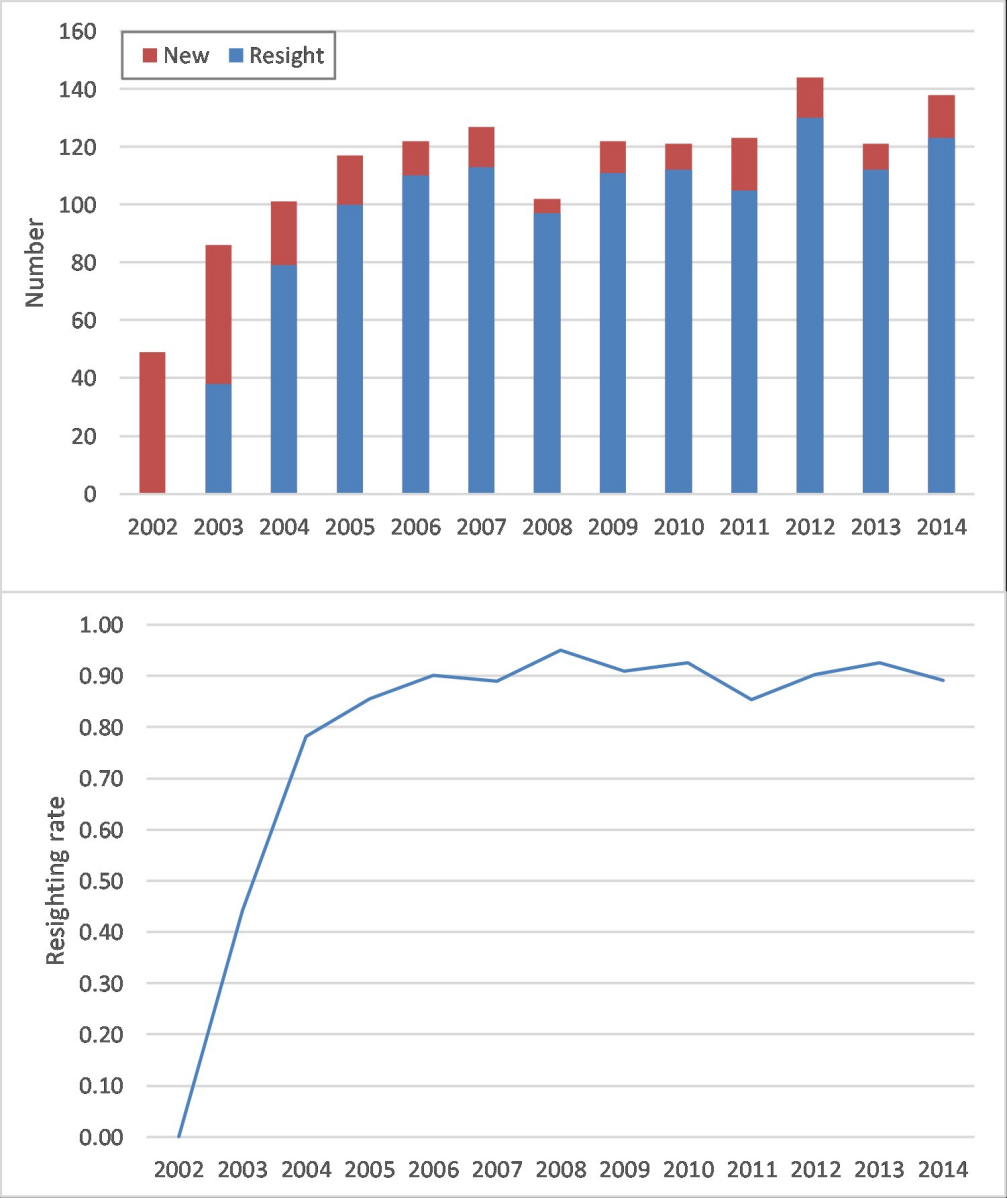
(a) The annual number of new and resighted gray whales and (b) the annual resighting rate (resighted individuals / total number of sighted individuals (new + resight)).

A total number of 243 individual gray whale utilized the feeding grounds off Sakhalin at some point during the period 2002–2014. The total number of individuals was compromised of 94 calves and 149 non-calves (Table 1). A total of 25 reproductive females were seen on the Sakhalin feeding grounds. The first reproductive female was identified around 10 years of age, as a calf seen in 2004 returned with her own calf in 2014. The discovery curve demonstrated that in the first few years of the study, the number of new non-calf whales encountered decreased rapidly and the discovery curve started to plateau after the first 400 sightings, equivalent to approximately the first 3 years of the study (2002–2004) (Figs 2 and 3). In fact, 119 out of the 149 (80%) new non-calf individuals observed in 2002–2014 were photographically captured within the first 4 years of survey effort (Fig 2). After these initial 4 years of photo-identification efforts, only 30 new non-calf individuals were observed in a span of 9 years (2006–2014). Thus, the majority of the newly identified individuals on the Sakhalin foraging grounds after 2005 were largely a result of calf production (72.0%) as opposed to new non-calf individuals (28.0%).

### Sightings within the nearshore and offshore feeding areas

Survey effort in the nearshore and offshore feeding areas was variable with more effort in the nearshore area in 2003–2014 (Table 2). This was due to both an increased amount of vessel-based effort in the nearshore area, as well as shore-based photo-identification efforts.

**Table 2:** Summary of photo-identification effort within the Sakhalin feeding areas.

	NEARSHORE AREA			OFFSHORE AREA		
Year	No. of surveys	No. of observation days with sightings	Total sightings	No. of surveys	No. of observation days with sightings	Total sightings
2002	8	5	17	17	8	60
2003	14	13	111	21	13	66
2004	24	31	258	3	4	7
2005	54	43	494	2	5	8
2006	22	43	458	4	8	60
2007	47	58	644	8	16	207
2008	22	38	218	8	6	99
2009	28	38	381	3	4	48
2010	22	48	458	5	7	24
2011	20	21	335	—	6	16
2012	20	18	203	14	15	168
2013	15	10	116	5	2	88
2014	28	44	573	10	5	123
TOTAL	324	410	4266	100	99	974

‘No. of surveys’ relates to dedicated inflatable boat-based surveys only, and does not include shore-based surveys or sightings by the large opportunistic vessel. ‘No. of observation days with sightings’ includes days with one or more dedicated surveys as well as days with large opportunistic vessel and/or shore-based sightings. — = no effort data available.

Nearly all the identified Sakhalin whales (239 out of 243) were observed in the nearshore area at some point during the 13 years of our study. A total of 122 individuals were identified in the offshore area and 118 individuals were seen in both feeding areas. Four individuals were observed only in the offshore area. These four individuals were sighted once and were never seen again in subsequent years. A total of 121 individuals were seen only in the nearshore area and never observed in the offshore area, of which 67 whales (including 44 calves) were seen only once. This included 15 whales (12 calves) that were first seen in our last year of observations (2014).

The total number of sightings, annual number of observed individuals and total number of unique observed individuals were significantly higher in the nearshore area than in the offshore area (χ^2^ = 2068.2, *p* < 0.001; χ^2^ = 231.1, *p* < 0.001; χ^2^ = 37.9, *p* < 0.001, respectively). Survey effort in the nearshore was 3–4 times more compared to the offshore area (Table 2). To correct for survey effort, the minimum number of sightings in a year in either the nearshore or the offshore area was used for both regions. This standardization of the number of sightings resulted in a total of 931 sightings of individuals in both the offshore and nearshore areas across all years (Table 3). The sum of the total number of individuals identified every year in these 931 sightings was not significantly different between the nearshore and offshore area (540 vs. 512; χ^2^ = 0.75, *p* <0.38), suggesting that sampling heterogeneity was similar in both survey areas. After the correction for effort, the total number of unique individuals in the nearshore and offshore areas was found to be different (200 vs 119; χ^2^ = 20.6, *p* <0.001). This indicates that the nearshore area was used by a larger number of individuals compared to the offshore area, and the difference was not due to an increase amount of effort within the nearshore area.

**Table 3:** Uncorrected number of sightings, individuals and cumulative number of unique individuals in the nearshore and offshore area, as well as the number of individuals and cumulative number of individuals in the nearshore and offshore area after standardizing for the number of sightings in both areas.

	Uncorrected						Corrected				
	Nearshore			Offshore			Total sightings	Nearshore		Offshore	
	Total sightings	No. ind	Cum. Ind	Total sightings	No. ind	Cum. Ind		No. ind	Cum. Ind	No. ind	Cum. Ind
2002	17	14	14	60	37	37	17	14	14	14	14
2003	111	67	74	66	33	51	66	50	58	33	41
2004	258	100	115	7	6	54	7	7	61	6	45
2005	494	116	134	8	7	55	8	8	65	7	46
2006	458	111	146	60	33	65	60	26	78	33	58
2007	644	107	159	207	68	84	207	113	94	68	81
2008	218	67	164	99	61	89	99	48	120	61	86
2009	381	102	175	48	39	92	48	36	129	39	89
2010	458	116	184	24	21	96	24	19	139	21	93
2011	335	115	202	16	13	97	16	15	143	13	94
2012	203	95	217	168	74	106	168	88	173	74	103
2013	116	68	224	88	67	114	88	57	185	67	111
2014	573	83	239	123	76	122	123	59	200	76	119
N	4266	1161		974	535		931	540		512	

‘Total sightings’ = total number of individuals seen during the year—includes multiple sightings of a single individual, ‘No. ind.’ = number of unique individuals seen during the year, ‘Cum. Ind.’ = cumulative number of individuals seen during the survey period.

### Site fidelity and residency times

Site fidelity to the Sakhalin feeding ground was found to be relatively high. For example, 10 of the 49 whales (20.4%) seen in Sakhalin in 2002 were sighted every year. Of these 49 whales, 38 (77.6%) were seen during 10 or more years out of the 13 years of photo-ID effort. Out of the 187 animals that could have been observed 5 years or more (i.e. first observed in period 2002–2010), 19.7% (n = 37) returned to either study area every year after the year of first sighting.

The mean annual return rates for the study period indicated that of all new individuals seen in a given year, on average over half of those new individuals (64.9%) were seen each year thereafter (Table 4A). In the first two years of survey effort (2002–2003), the mean annual return rates were higher (77.3%) compared to other years (Table 4A). This may be explained by a larger proportion of newly identified non-calf animals vs. calves. A relatively high number of observed calves were sighted only once and not again (35 excluding calves seen in 2014). Excluding those 35 calves seen only once resulted in higher mean annual return rates, i.e. 72.2% vs 64.9% (Table 4B).

**Table 4:** Annual return rates of WNP gray whales defined by the total number of unique individuals identified/year and the number of animals resighted in subsequent years.

(a) Year	N	2003	2004	2005	2006	2007	2008	2009	2010	2011	2012	2013	2014	Mean (n)	Mean (%)
2002	49	38	42	44	46	43	40	42	39	30	39	27	36	38.8	79.3
2003	48	-	37	39	39	40	35	39	34	34	37	33	30	36.1	75.2
2004	22		-	17	14	13	12	13	13	8	13	12	12	12.7	57.7
2005	17			-	11	9	5	8	10	10	11	9	8	9.0	52.9
2006	12				-	8	4	5	4	5	7	3	6	5.3	43.8
2007	14					-	1	3	3	3	2	2	1	2.1	15.3
2008	5						-	1	1	1	1	1	0	0.8	16.7
2009	11							-	8	8	7	5	8	7.2	65.5
2010	9								-	6	6	6	5	5.8	63.9
2011	18									-	7	7	6	6.7	37.0
2012	14										-	7	7	7.0	50.0
2013	9											-	3	3.0	33.3
2014	15												-	n/a	n/a
	243														64.9
(b) Year	N	2003	2004	2005	2006	2007	2008	2009	2010	2011	2012	2013	2014	Mean (n)	Mean (%)
2002	49	38	42	44	46	43	40	42	39	30	39	27	36	38.8	79.3
2003	46	-	37	39	39	40	35	39	34	34	37	33	30	36.1	78.5
2004	21		-	17	14	13	12	13	13	8	13	12	12	12.7	60.5
2005	16			-	11	9	5	8	10	10	11	9	8	9.0	56.3
2006	9				-	8	4	5	4	5	7	3	6	5.3	58.3
2007	5					-	1	3	3	3	2	2	1	2.1	42.9
2008	1						-	1	1	1	1	1	1	1.0	100.0
2009	9							-	7	8	7	5	8	7.0	77.8
2010	9								-	6	6	6	5	5.8	63.9
2011	10									-	7	7	6	6.7	60.6
2012	12										-	7	7	7.0	58.3
2013	6											-	3	3.0	50.0
2014	15												-	n/a	n/a
	208														72.2

(a) = Including 35 calves that were seen one year and not in following years. (b) = excluding 35 calves that were seen one year and not in following years. ‘Year’ = original sighting year, ‘N’ = number of new individuals sighted in original sighting year, ‘2003–2014’ = resighting years.

**Fig 4:**
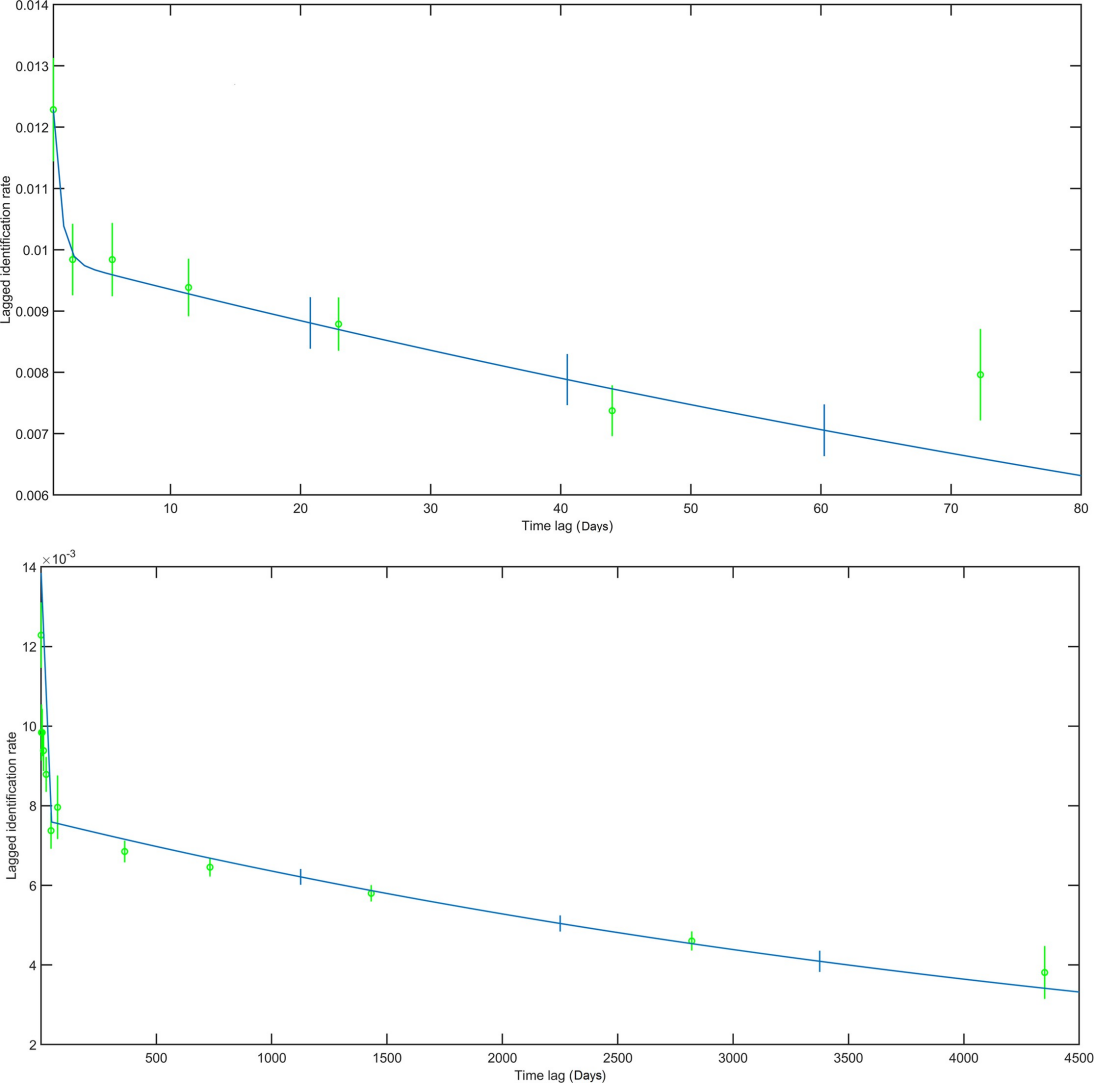
LIR for a single season (top) and for the total study duration (2002–2014) (bottom). The green circles provide the LIR of different lagged time bins.

The annual resighting rate, i.e. the proportion of individuals sighted during a year that were seen in previous years, was high as well, and leveled off after the first three years of observation (Fig 4). After these first three years the mean rate of annual return was on average 90%, meaning that approximately 9 out of 10 animals sighted during a year have been seen in previous years.

### Area usage

#### Total area

The average LIR over a season dropped abruptly within the first few days and subsequently decreased gradually over the course of a season, with a small increase after 70 days (Fig 4 top). Lagged identification rates did not reach zero over the course of a season suggesting that the period of observations did not cover the entire feeding season. The usual start of observations occurred after the first whales arrived on the feeding grounds and stopped before all animals departed for their wintering grounds. For these reasons the maximum residency times could not be assessed. LIR rates over the survey period 2002–2014 saw a decrease within the first year, followed by a subtle lag period of 13 year (Fig 4 bottom). Based on the QAIC criteria, models 7 (emigration + reimmigration) and 8 (emigration + reimmigration + mortality) were selected for the annual (χ^2^ = 93.4, d.f. = 69, *p* < 0.05) and multi-year LIR (χ^2^ = 1529.8, d.f. = 1320, *p* < 0.01), respectively. The selected model for the annual and multi-year LIR was 92 and 91 individuals, respectively, present on the Sakhalin feeding grounds at any given time during the feeding season. The mean number of days of whales remaining on the Sakhalin feeding grounds compared to outside of the Sakhalin feeding grounds during the feeding season was estimated to be 72.0 and 41.9 days for the annual season and 64.3 and 32.6 days for the multi-year data set (S2 Table). These figures may have been an underestimation as no data were collected early during the feeding season (mid-May—June) and later in the season (October—December). Multi-year LIR for animals first seen as calves are provided in S1 Fig.

**Fig 5:**
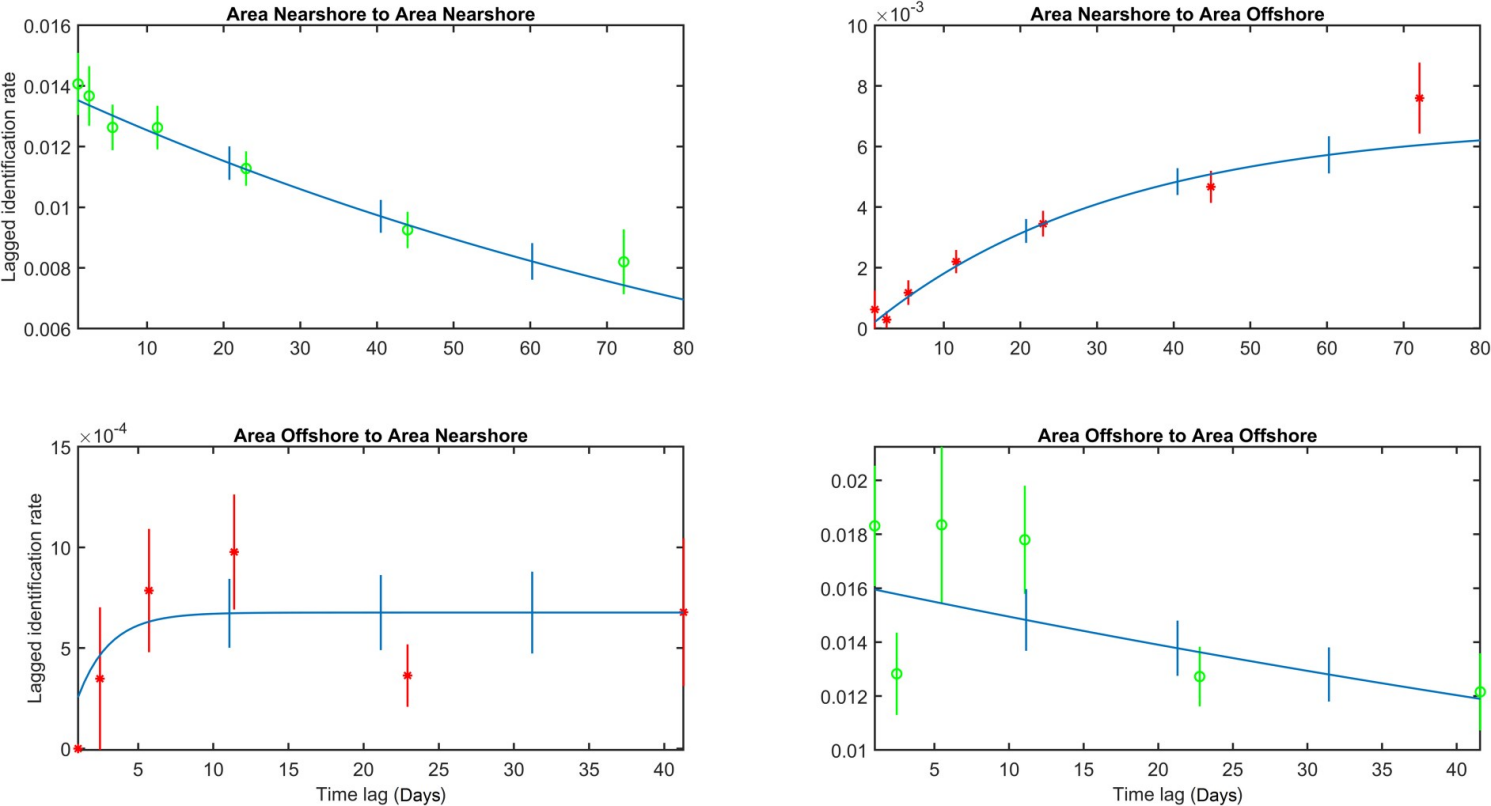
LIR and selected models within and between the offshore and nearshore area within a season. Red stars and green circles are the LIR of different lagged time bins.

*Seasonal movement between nearshore and offshore area*. LIR between the ‘nearshore to nearshore’ area (i.e. the likelihood that a random individual first seen in the nearshore area was sighted there again) decreased over the course of a field season but did not reach zero, indicating that some, but not all, animals left this area (Fig 5 top-left). Over this same period, a continuous increase in LIR values between the ‘nearshore into offshore’ area was observed, suggesting a movement of individuals from the nearshore to the offshore area over the season (Fig 5 top-right). The likelihood of seeing individuals in the offshore area first and consequently in the nearshore area was low but increased for the first 10 days suggesting some movement back and forth between both feeding areas (Fig 5 bottom-left). There was a decrease in LIR values in the ‘offshore to offshore’ model after 10–20 days, after which the LIR remained constant between 20–40 days (Fig 5 bottom-right). These LIR values were high in comparison to the ‘offshore to nearshore’ LIR values, suggesting that more animals stayed in the offshore area instead of moving to the nearshore area. Furthermore, ‘offshore to nearshore’ LIR values were low in comparison to ‘nearshore to offshore’ values which is indicative of more animals moving from the nearshore to the offshore area, instead of the other way around.

The maximum lag for the offshore to offshore area for which LIR values could be obtained was 40 days, which was noticeably less than the maximum lag of the nearshore to nearshore area (>70 days). This was likely due to reduced survey effort in the offshore area, with less time between the first and last surveys over a season. Details of the chosen models were included in S3 Table. The selected models fit did have anomalies in the estimated number of animals and mean residence times. For that reason, these values were not considered.

#### Multi-annual movement between nearshore and offshore area

**Fig 6:**
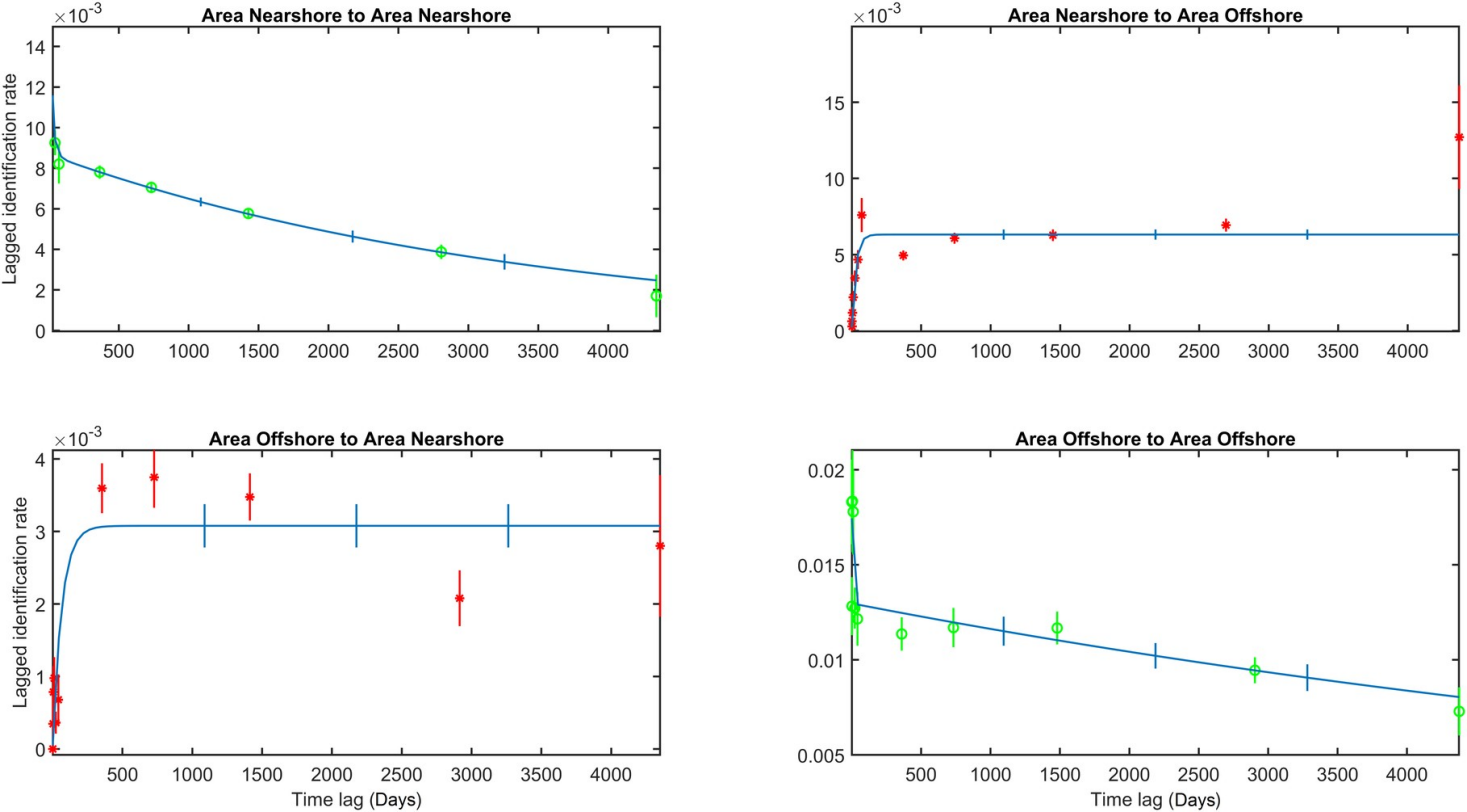
LIR within and between offshore and nearshore area over the duration of the study period (2002–2014). Red stars and green circles are the LIR of different lagged time bins.

Lagged identification rates between the nearshore to the offshore area increased over the course of the first season and then gradually increased from year two up to a lag period of 7–8 years (Fig 6 top-right). This means that once animals start migrating to the offshore area during a season, most of them continued to do so consistently in following seasons. Of specific interest was the steep increase in LIR after 12 years, with LIR values twice as high compared to after one year. These results suggest that especially mature animals (>12 years) utilized the offshore feeding area more frequently and younger animals less frequently or not at all, as is the case with calves and yearlings. (. In fact, only 11 of the 94 calves (12%) observed in the nearshore area during 2003–2014 were also seen on the offshore feeding area during later years. The average period between the first sighting of a calf in the nearshore area and the first sighting of that individual in the offshore area for these 11 individuals was about 5 seasons (4.8 ± 1.85). Of the 122 individual Sakhalin whales never observed in the offshore area, 83 (68%) were first seen as calves, 19 (16%) were non-calves that were seen only once on the nearshore area and not again, 6 (5%) were reproductive females which were known to prudentially utilize the nearshore area and 14 (11%) did not fit in these three categories. This indicated a demographical difference in area usage with younger animals more frequently utilizing the nearshore area, and increased use of the offshore area by older animals.

As previously mentioned, there was limited movement between the offshore to the nearshore area within the first year of observation in the offshore area (Fig 6 bottom-left). However, increased LIR values for lags of 2–8 years indicated that animals seen in the offshore area moved consistently to the nearshore area in following years (Fig 6 bottom-left). After 8–12 year, LIR values decreased suggesting reduced use of the nearshore area with increased maturity. This finding corresponds with the above-mentioned high ‘nearshore to offshore’ LIR values for mature animals. LIR values for the ‘offshore to offshore’ area for lags larger than a year (Fig 6 bottom-right) appeared more constant and decreased less in comparison with LIR values for ‘nearshore to nearshore’ for similar lags (Fig 6 top left), suggesting higher site fidelity to the offshore area. The model output was not further considered as the model fits were either poor, or had anomalous estimates for animals present on the feeding grounds and residency times. LIR trends within and between the nearshore and offshore areas for the period 2002–2014, based on animals that were first seen as calves (S2 Fig top-right), had somewhat similar trends as those for based on all individuals (Fig 6). The LIR values for ‘nearshore area to offshore area’ for calves only showed a more profound increase compared to that based on all individuals. One other difference was that there was no negative or positive trend observed in the ‘offshore to offshore area’ LIR based on animals first seen as calf, as was the case in that based on all individuals (S2 Fig bottom-right). This suggests that once the few young animals move the offshore area, they consistently return to this area in following years.

## Discussion

Photo-identification is an important methodological technique that provides a means to understand individual spatiotemporal movement patterns of gray whales. The gray whale is a suitable species for photo identification studies as individuals are born with unique and persistent pigmentation patterns and display consistent scarring and ectoparasites patterns [86–88]. Although scarring and parasite patterns can change over the years, individuals can typically be identified with certainty as these patterns change slowly and most animals are seen consistently over the year(s), providing a chronological photo record. Natural pigmentation, ectoparasites and scarring can also be used to reliably distinguish calves from yearlings, even in the absence of their mothers [86].

Off Sakhalin, a greater understanding of WNP gray whales’ utilization, movement and site fidelity within their foraging habitat facilitates the development of new, or adjustment of existing, conservation management efforts of this population [89]. For example, offshore oil and gas activities in the vicinity of the nearshore feeding area have the potential to impact the WNP population and require detailed mitigation strategies [47, 48]. Lastly, quantification of spatiotemporal movement parameters is also needed to better assess the potential population level consequences of disturbance in specific risk assessment frameworks [49, 50]. This is especially the case when bioenergetic models are used that require information on the energetic cost of movement patterns over the available feeding areas and difference in prey availability in these areas [49, 50].

Whales feeding on the Sakhalin foraging grounds consistently returned annually during this 13-year study period. The discovery curve plateaued after the initial three years of photo-identification effort with further increases of new individuals being primarily a result of reproduction. This is an indication that the WNP gray whale population is a closed population with little to no immigration at least on their foraging grounds. The 31 new non-calf whales observed in the period 2006–2014 could either be new individuals that immigrated from the ENP population or calves of the WNP population that this photo-identification study was unable to capture as a calf, which is known to occur at times. Similarly, Weller *et al*. [20] found that the discovery curve plateaued after 4 years of survey effort of gray whales off Sakhalin. The spatial area of our surveys covered a larger area which included the entire nearshore and offshore areas and demonstrates that there was little immigration of new adults to the Sakhalin feeding grounds. This result is also consistent with population models conducted by Cooke *et al*. [14, 90] who concluded that the Sakhalin gray whale population is demographically self-contained with growth rates being exclusively a product of calf production and little immigration into the population. One presumably female whale with a calf was known to be 10 years old since this whale was sighted as a calf. This observation is in line with other observations of gray whale reaching age of sexual maturation between the ages of 5 and 12 [91, 92].

The formation of "feeding groups" that show high site fidelity is not uncommon among gray whales. For example, a small group of ENP gray whales (the Pacific Coast Feeding Group) has consistently been observed to feed between the Californian coast and the Alaskan peninsula [93, 94]. Another example is by Heide-Jørgensen *et al*. [36], who observed high fidelity in satellite-tracked ENP gray whales to relatively small-scale summering feeding grounds off Chukotka, Russia, as well. High site fidelity to feeding areas is not always observed in baleen whales, with daily feeding trips exceeding 100 km/day in some species such as blue whales (*Balaenoptera musculus*), fin whales (*Balaenoptera physalus*) and minke whales (*Balaenoptera acutorostrata*) [63–65], but can occur in some other baleen whales species, such as humpbacks and bowhead whales [36, 68, 95]. Gray whales are unique among baleen whale as they are true (epi)benthic foragers, although bowhead whales can target dense prey concentrations near or on the seabed as well [68]. (Epi)benthic foragers are more likely to show high site fidelity to specific feeding grounds where there are predictable, high density, but spatially restricted, prey concentrations [36]. Given the large energy needs of these large whales, they likely have evolved to exploit their prey in regions with high density aggregations [68].

### Area usage

A closer examination of the area usage within the Sakhalin feeding grounds within and between years indicated a higher area usage of the nearshore area versus the offshore area, with more individuals making use of the nearshore area in comparison to the offshore area. Three possible explanations, which are further described below, include: 1) biased survey effort (i.e., higher survey effort in the nearshore area), 2) availability of more accessible (i.e. shallower) food resources in the nearshore area, and 3) demographical differences in area use. Since about 76% of our survey effort occurred in the nearshore area, the high degree of area usage here compared to the offshore area could have been a result of effort bias, but after correcting for survey effort, more individuals were still seen in the nearshore area. Although more individuals were seen in the nearshore area, lagged identification rates suggested a higher site fidelity to the offshore area, especially when animals start to mature.

Gray whales are known to be opportunistic feeders, taking advantage of changes in food availability [51, 52, 96]. Bluhm *et al*. [56] suggested that gray whale area usage is linked to high prey density, which has been seen in other baleen whale species as well [97]. This was thought to be a more important explanatory factor for gray whale density than the taxonomic composition of the available prey. The prey resources in the nearshore area are mainly comprised of smaller amphipods (*Monoporeia affinis*) distributed in aggregated patches that vary significantly in biomass concentration from year to year [22], but include other prey species as well [73]. In contrast, the offshore area is comprised of a high concentration of ampeliscid amphipods (*Ampelisca eschrichtii*), with a mean biomass of 338.2 gr/m^2^ [21, 57]. The offshore area is deeper (40-60m) and therefore potentially more energetically demanding than the nearshore area (<20 m), but also likely to be more energetically rewarding due to this high biomass of prey availability.

A third possible explanation is that mother-calf pairs, and young animals, prefer the shallow near shore waters over the offshore area. In the 13 years of offshore feeding area surveys no calves, mother-calf pairs or yearlings have been observed in the offshore area, and only 12% of calves (11) seen between 2003–2014 were observed in the offshore area. Based on shore-based behavioral studies in the nearshore feeding area, it is known that mother-calf pairs and weaned calves in the nearshore feeding area have significantly longer respiration intervals and shorter dive times compared to other individuals (any individual excluding mothers, calves and yearlings) [98]. Furthermore, even within the nearshore feeding area there is spatial age-class segregation as observed by significant differences in the mean distance to shore between mother/calf pairs (0.55 km), weaned calves (0.81 km) and other individuals (1.30 km) [98]. The relatively shallow depths in the near shore feeding area compared to the offshore area, is likely to make it easier for a mother-calf pair to access prey resources. Nearshore affinity of gray whale mother-calves on breeding and feeding grounds, as well as during migration, has been observed in several studies [98–101]. Another possible reason for avoidance of the offshore area by calves, yearling and young animals is predator avoidance behavior. Transient killer whales have been observed each feeding season to traverse through their foraging grounds off northeastern Sakhalin. A high percentage of killer whale tooth scars were found on WNP gray whales compared to similar estimates for other baleen whale species [20, 102]. Little interaction between the two species has been observed off Sakhalin, however, but killer whale attacks, both of calves and adults, are common in certain areas off California, Alaska and Chutoka, Russia [102]. For example, the risk of predation of gray whales by killer whales was studied over a 10-year period along Chukotka by Melnikov and Zagrebin [103] who found that two-thirds of approximately 100 reported killer whale attacks on marine mammals were on gray whales. The shallow nearshore area may provide more shelter or avoid detection due to the noisy surf region compared to the offshore area, although the specific mechanism is not well understood. Gray whales off California are observed to ‘hide’ from killer whales behind rocks or in kelp beds [104]. It has been hypothesized that kelp could offer a physical and an acoustic screen for protection through possible interference with echolocation used by killer whales either by the gas-filled pneumatocysts, or canopy and surf noise [104]. The nearshore feeding area lacks rock formations and kelp beds, but presence of surf noise could make acoustic detection by killer whales more difficult. We therefore hypothesize that younger animals make more use of the nearshore feeding season because of a combination of easier prey availability and reduced risk of killer whale attacks. With increasing age, mature animals may be better able to access the deeper and abundant prey of the offshore area needed to sustain their increased energy requirements [50, 105].

### WNP population size

In the early 1970s, the WNP gray whale population was thought to be extinct as a result of over-exploitation from commercial whaling, until a few gray whales were seen in the Sea of Okhotsk [5–7]. Since then there has been an intensive effort to study the population size, health, and origin of these gray whales [20, 76, 90, 106]. Mark-recapture estimates of WNP gray whales population size yielded a non-calf estimate (with 90% Bayesian confidence intervals) of 122 (CI = 113–131) individuals in 2006 [107], 121 (112–130) non-calf individuals in 2007 [108], 130 (120–142) non-calf individuals in 2008 [109], 140 (±6) non-calf individuals in 2012 [90] and 174 (158–191) to 186 (171–203) non-calf individuals in 2015 [14] (depending on whether individuals last seen as calves were considered to have died or merely left). In our study a total of 82 calves were identified in the period 2003–2013 of which 35 animals (43%) were not resighted after the first year of observation. It is not known whether these calves died or just did not return to the Sakhalin feeding ground. Considering the high site fidelity expressed by Sakhalin gray whales and low survival rates observed during calves’ first year of life [45, 110], it is more likely these animals died. Low calf survival rates for baleen whale calves is not uncommon and observed in, for example, ENP gray whales [10, 111] and humpback whales as well [112]. In the total study period (2002–2014) a total of 243 individuals were observed, with identification of a total of 184 individuals (or 172 excluding 12 calves seen in 2014) in the last three years of this period (2012–2014). This number is within the Cooke *et al*. [14] estimated range.

### Site fidelity and movement patterns

LIR values decreased during the first season of observation and reduced gradually over the following survey years due to emigration and/or mortality, indicating that animals return consistently to the Sakhalin feeding area. Some animals in the nearshore area were there only for part of the feeding season, with gradual migration to the offshore area over the remainder of the season, probably driven by availability of food resources. Other animals in the nearshore area, mostly calves or young animals, but also 6 out of 25 reproductive females, did not move to the offshore area. Individuals in the offshore area displayed higher within-season site fidelity in comparison to the nearshore feeding area, with only limited movement to the nearshore area. However, animals seen in the offshore area were consistently seen in the nearshore area in future years. They seemed to follow an annual cycle of first arriving on the nearshore feeding area with movement to the offshore area during the latter part of the feeding season. Use of the nearshore area decreases with increasing lagging rates (8–12 years).

The fact that within-season LIR values did not approach zero, and remained fairly constant after the maximum observed within-season lag period of 70–80 days, was indicative of insufficient duration of the survey period to fully capture the migration towards wintering grounds. The nearshore feeding area was typically sea ice-free after June [45], although individuals can arrive on the foraging grounds before ice-free conditions [30, 45], and photo-identification field seasons mostly started in July. Survey efforts ended around the beginning of October due to adverse weather conditions often prevailing in this period. Migration towards the breeding grounds is characterized by temporal segregation of different age, sex and reproductive classes, with pregnant females migrating first, followed by non-pregnant females, adult males and immatures [87, 101]. Based on individuals that were satellite tagged, onset of migration of at least three non-pregnant individuals started towards the end of November up to mid-December [30]. Whereas some demographic classes may have started migration before then, e.g. pregnant females, these findings confirm that photo-identification efforts did not cover the entire feeding season.

The slow and gradual decrease in multi-year LIR value demonstrates high site-fidelity to the feeding grounds off Sakhalin, i.e. the likelihood of sighting a specific individual after the first year of observation did not decrease much over the 13 years of observation (Fig 4). The observed small decrease in LIR starting after a lag of one year can be explained by emigration and mortality, as well as the growth in population size, which reduces the likelihood of resighting a specific individual. LIR rates between the nearshore and offshore areas demonstrated a constant exchange between these two feeding areas. As mentioned previously, a gradual increase of nearshore mature individuals moving into the offshore area over a feeding season was detected, which may be explained by reduced prey levels in the shallower feeding ground over the season, warranting feeding in deeper waters with high prey biomass. These results were consistent by the study of Heide-Jørgensen *et al*. [36] who also found gray whale affinity to relatively shallow areas (<30m) off Chukotka, Russia, and an increased use of deeper areas (70m) later during the feeding season. Although some mature animals frequent the offshore area in the later part of the feeding season, LIR values indicated that these individuals typically arrive in the nearshore area early during the next feeding season, followed by the move to the offshore area later in the season. The steep increase at nearshore to offshore LIR values at 13 years demonstrates an increased preference of mature animals to the offshore area, which could be related to the higher energetic demands of the larger animals [105], an enhanced ability to feed in deeper waters or a reduced risk of killer whale predation. This type of age class spatial segregation is commonly observed in gray whales, not just during the feeding season but also during migration and on the breeding grounds [36, 87, 99, 101, 104].

The combination of (i) high site fidelity of WNP gray whales to the nearshore and offshore feeding ground, (2) absence of other known feeding areas in the Sea of Okhotsk, and (3) the relatively small spatial area of the nearshore and offshore feeding areas, makes the WNP population more susceptible to environmental changes and/or anthropogenic disturbance such as fisheries or oil & gas development. Despite these factors this WNP gray whale population has seen an average growth rate of 4.3% between 2005 and 2015 [14], but warrants continued focus to ensure this upward trend continues.

Photo-identification is an important methodological technique that provides a means to understand individual spatiotemporal movement patterns of gray whales. Off Sakhalin, a greater understanding of WNP gray whales’ utilization, movement and site fidelity within their foraging habitat facilitates the development of new, or adjustment of existing, conservation management efforts of this population [89]. For example, offshore oil and gas activities in the vicinity of the nearshore feeding area have the potential to impact the WNP population and require detailed mitigation strategies [47, 48]. Lastly, quantification of spatiotemporal movement parameters is also needed to better assess the potential population level consequences of disturbance in specific risk assessment frameworks [49, 50]. This is especially the case when bioenergetic models are used that require information on anthropogenic or natural factors affecting energy intake, energetic cost of movement patterns over the available feeding areas and difference in prey availability in these areas [49, 50]. In fact, interannual variation in the energy reserves of whales has been detected and correlated with both prey availability and whale fecundity [113], as well as with sea spring ice cover [45]. During years with more sea ice cover or lower prey availability in the nearshore feeding area, animals might make more use of the offshore area to compensate. An enhanced understanding of these processes and movement patterns are therefore relevant for purpose of conservation management.

## Conclusions

This long-term photo-identification study identified that most of the WNP gray whale population consistently returned to their summer foraging grounds in the Sea of Okhotsk on an annual basis. WNP gray whales display a high degree of site fidelity to both feeding areas off Sakhalin. More WNP gray whales make use of the nearshore feeding area, in comparison to the offshore area. This is especially the case during the early years of individuals, which may be due in part to more easily accessible food resources and a more protective environment from predators for young animals. There is a consistent seasonal and multi-year movement between the nearshore and the offshore feeding areas. This includes more seasonal movement of mature individuals from the nearshore into the offshore feeding area over the course of a season, and eventually reduced usage of the nearshore feeding area. The long-term nature of our photo-identification dataset, high rate of annual return of individuals to the feeding grounds, and the consistent trend of observing most “new” individuals as calves (as opposed to adults), along with little evidence of immigration allows us to conclude that the gray whale population returning annually to Sakhalin waters is demographically self-contained. The nearshore and offshore Sakhalin feeding grounds are therefore important areas for summer feeding of young individuals and mothers, and mature animals, respectively.

## Supporting information

S1 Fig LIR for total study area and study duration (2002–2014), based on individuals that were first observed as a calf. (TIF)

S2 Fig LIR within and between offshore and nearshore area over the duration of the study period (2002–2014), based on individuals that were first observed as a calf. (TIF)

S1 Table Models run for lagged identification rates. Models run as preset in SOCPROG 2.7 (Whitehead, 2009). Parameters test for population closure (1 and 2), as well as emigration, reimmigration, and mortality rates (3 to 8). The quasi-Akaike Information Criterion was used for goodness of fit.
(DOCX)

S2 Table Selected models for lagged identification rates for the Sakhalin feeding grounds, including no. of selected model, p-value, estimated number of individuals and mean residence times. (DOCX)

S3 Table Selected models for lagged identification rates between and within nearshore and offshore areas, including no. of selected model, p-value, estimated number of individuals and mean residence times. (DOCX)
