# Supplementary figures and images for: Site-fidelity and spatial movements of western North Pacific gray whales on their summer range off Sakhalin, Russia
Source: PLoS One. 2020 Aug 14;15(8):e0236649. doi: 10.1371/journal.pone.0236649 (PMC7428188; doi:10.1371/journal.pone.0236649)

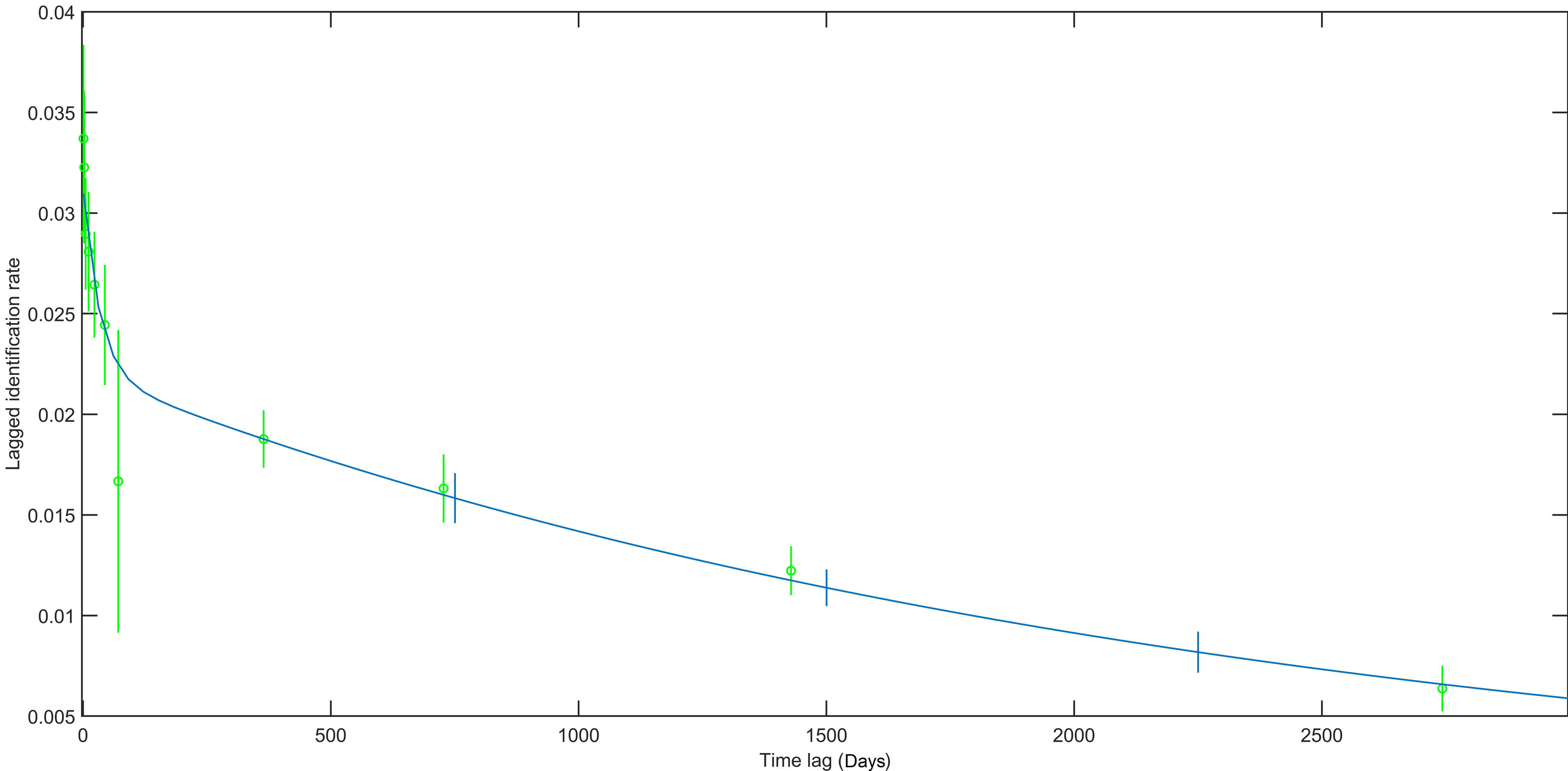

Supplement: S1 Fig — (TIF) [file pone.0236649.s001.tif]

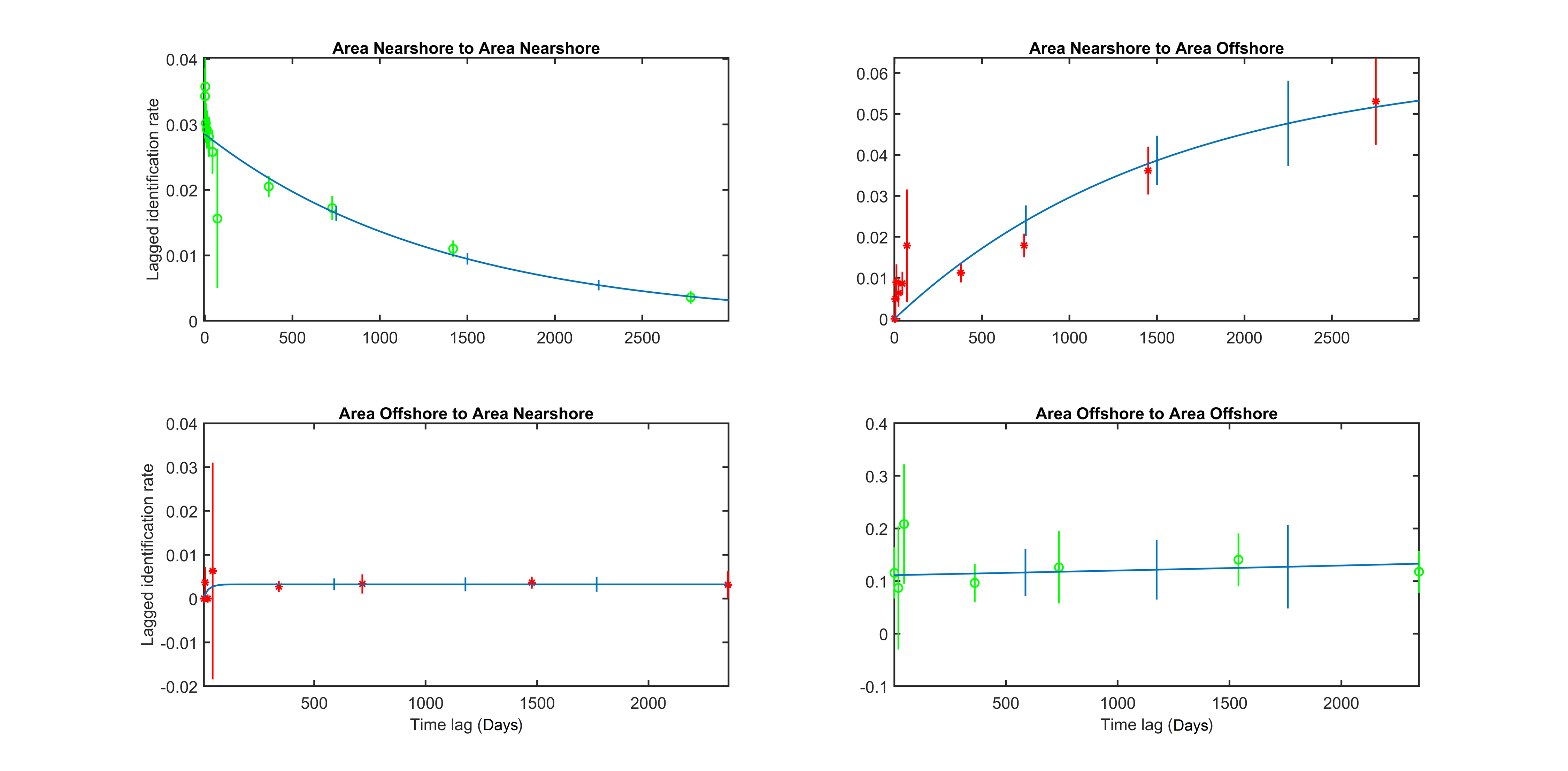

Supplement: S2 Fig — (TIF) [file pone.0236649.s002.tif]
