# Supplementary material for: Site-fidelity and spatial movements of western North Pacific gray whales on their summer range off Sakhalin, Russia
Source: PLoS One. 2020 Aug 14;15(8):e0236649. doi: 10.1371/journal.pone.0236649 (PMC7428188; doi:10.1371/journal.pone.0236649)
Supplement: S1 Table — Models run as preset in SOCPROG 2.7 (Whitehead, 2009). Parameters test for population closure (1 and 2), as well as emigration, reimmigration, and mortality rates (3 to 8). The quasi-Akaike Information Criterion was used for goodness of fit. (DOCX) [file pone.0236649.s003.docx]

S1 Table 1 Models run for Lagged Identification Rates. Models run as preset in SOCPROG 2.7 (Whitehead, 2009). Parameters test for population closure (1 and 2), as well as emigration, reimmigration, and mortality rates (3 to 8). The quasi-Akaike Information Criterion was used for goodness of fit.

| **Name** | **Explanation** | **Equation** |
| --- | --- | --- |
| With one study area (N is population size in study area): | | |
| 1 | Closed (1/a1 = N | a1 |
| 2 | Closed (a1 = N) | 1/a1 |
| 3 | Emigration/mortality (a1 = emigration rate; 1/a2 = N) | a2*exp(-a1*td) |
| 4 | Emigration + reimmigration (a1=emigration rate; *a2/(a2* + *a3)* = proportion of population in study area at any time) | (1/a1)*exp(-td/a2) |
| 5 | Emigration/mortality (a1 = N; *a2* = *Mean* residence time) | a2+a3*exp(-a1*td) |
| 6 | Emigration + reimmigration + mortality | (1/a1)*((1/a3)+(1/a2)*exp(-(1/a3+1/a2)*td))  /(1/a3+1/a2) |
| 7 | Emigration + reimmigration (a1 = N; *a2* = *Mean* time in study area; *a3* = *Mean* time out of study area) | a3*exp(-a1*td)+a4*exp(-a2*td) |
| 8 | Emigration + mortality + reimmigration (a1 = N; *a2* = *Mean*  time in study area; *a3* = *Mean* time out of study area;  *a4* = *Mortality* rate | (exp(-a4*td)/a1)*((1/a3)+(1/a2)*exp(-(1/a3+1/a2)*td))/(1/a3+1/a2) |
| With two study areas (N is total population size): | | |
| 1 | Fully mixed (1/a1=N) | a1 |
| 2 | Fully mixed (a1=N) | 1/a1 |
| 3 | Migration—full interchange (a1=diffusion rate from area 1 to area 2; a2=1/N) | a2*(1-exp(-a1*td)) |
| 4 | Migration—full interchange (a1=N; a2=Mean residence time in area 1) | (1/a1)*(1-exp(-td/a2)) |
