# Supplementary material for: Site-fidelity and spatial movements of western North Pacific gray whales on their summer range off Sakhalin, Russia
Source: PLoS One. 2020 Aug 14;15(8):e0236649. doi: 10.1371/journal.pone.0236649 (PMC7428188; doi:10.1371/journal.pone.0236649)
Supplement: S2 Table — (DOCX) [file pone.0236649.s004.docx]

**S2 Table 1. Selected models for Lagged Identification Rates for the Sakhalin feeding grounds, including no. of selected model, p-value, estimated number of individuals and mean residence times.**

|  | Model selected | P | N | Mean res. time in | Mean res. time out | Mortality |
| --- | --- | --- | --- | --- | --- | --- |
| Single season | 7 | 0.027 | 92.2 | 72.0 | 41.9 | -- |
| Multiple season | 8 | 0.000 | 91.5 | 64.3 | 32.6 | 0.16x10^-3^ |
