# Supplementary material for: Site-fidelity and spatial movements of western North Pacific gray whales on their summer range off Sakhalin, Russia
Source: PLoS One. 2020 Aug 14;15(8):e0236649. doi: 10.1371/journal.pone.0236649 (PMC7428188; doi:10.1371/journal.pone.0236649)
Supplement: S3 Table — (DOCX) [file pone.0236649.s005.docx]

**S3 Table. Selected models for Lagged Identification Rates between and within nearshore and offshore areas, including no. of selected model, p-value, estimated number of individuals and mean residence times.**

|  | from | to | Model selected | P | N | Mean residence time | Mean res. time in | Mean res. time out |
| --- | --- | --- | --- | --- | --- | --- | --- | --- |
| Single season | Nearshore | Nearshore | 5 | 0.77 | 73.3 | 118.7 | - | - |
|  | Nearshore | Offshore | 4 | 0.44 | 147.7 | 32.3 | - | - |
|  | Offshore | Offshore | 5 | 0.71 | 62.3 | 138.1 | - | - |
|  | Offshore | Nearshore | 4 | 0.77 | 1479.8 | 2.1 | - | - |
| Multiple season | Nearshore | Nearshore | 8 | 0.07 | 65.6 | - | 50.9 | 38.6 |
|  | Nearshore | Offshore | 4 | <0.0001 | 158.3 | 28.7 | - | - |
|  | Offshore | Offshore | 8 | 0.99 | 41.2 | - | 2.3 | 2.0 |
|  | Offshore | Nearshore | 4 | <0.0001 | 324.9 | 64.9 | - | - |
